# Migration and Mobility: Correlates of Recent HIV Testing Among Substance Using Female Sex Workers at the Mexico–Guatemala Border

**DOI:** 10.1007/s10461-021-03501-8

**Published:** 2022-01-04

**Authors:** Teresita Rocha-Jiménez, Sonia Morales-Miranda, Carmen Fernández-Casanueva, Jay G. Silverman, María Luisa Zúñiga, Shira M. Goldenberg, Noe Crespo, Kimberly C. Brouwer

**Affiliations:** 1 Division of Infectious Diseases and Global Public Health, University of California San Diego, La Jolla, CA USA; 2 Society and Health Research Center, Faculty of Humanities, Universidad Mayor, Santiago, Chile; 3 Consorcio de Investigación Sobre, VIH SIDA TB CISIDAT, Cuernavaca, Morelos Mexico; 4 Centro de Investigaciones y Estudios Superiores en Antropología Social CIESAS, San Cristóbal de las Casas, Chiapas Mexico; 5 School of Social Work, San Diego State University, San Diego, USA; 6 Division of Epidemiology and Biostatistics, San Diego State, San Diego, CA USA; 7 School of Public Health, San Diego State University, San Diego, CA USA; 8 Department of Family Medicine & Public Health, University of California San Diego, La Jolla, CA USA

**Keywords:** HIV testing, Migration, Mobility, Sex work, Mexico–Guatemala

## Abstract

The goal of this paper is to determine the association between traveling to engage in sex work in another country and recent access to HIV testing among substance-using female sex workers (FSWs) in the Mexico–Guatemala border region. From 2012 to 2015, through modified time-location sampling and peer referral, 255 FSWs were recruited at Mexico’s southern border. Participants completed questionnaires on sociodemographics, migration and mobility experiences, work environment factors, and substance use. A conceptual framework, as depicted by a directed acyclic graph (DAG), guided our analysis. Crude and adjusted logistic regression models were used to evaluate the relationships between mobility experiences and HIV testing in the past year. Overall HIV testing was low (41%); after considering relevant covariates (i.e., interaction with health services and organizations, and sex work characteristics) traveling to engage in sex work in another country was found to be positively associated with HIV testing in the past year. Future efforts need to consider voluntary and non-stigmatizing prevention HIV services and focus on reaching out to less mobile women.

## Resumen

El objetivo de este artículo es determinar la asociación entre viajar a otro país para ejercer el comercio sexual y el acceso a una prueba reciente de VIH, en una población de mujeres trabajadoras sexuales en la frontera de México con Guatemala. Entre el 2012 y 2015, utilizando un muestreo por conveniencia y por referencia de pares, se invitó a 255 mujeres trabajadoras sexuales en la frontera sur de México a participar en este proyecto de investigación. Las participantes completaron una encuesta que comprendió preguntas sociodemográficas así como experiencias de migración y movilidad, características del trabajo sexual, y patrones de uso de sustancia. Utilizamos un marco conceptual que se ilustra en una Gráfica Acíclica Dirigida (DAG) el cual sirvió para guiar nuestro análisis. Realizamos análisis de regresión logística cruda y ajustada para evaluar la asociación entre experiencias de movilidad y haber recibido una prueba de VIH en el último año. En general, el porcentaje de haber recibido una prueba de VIH en el último año fue bajo (41%); después de considerar covariables relevantes; (ej., interacción con organizaciones y servicios de salud, características del trabajo sexual) encontramos que viajar a otro país para ejercer el comercio sexual está asociado con haber recibido una prueba de VIH en el último año. Es importante que, en el futuro, se concentren esfuerzos en servicios de prevención del VIH que sean voluntarios, no estigmatizantes, y que se enfoquen en mujeres trabajadoras sexuales menos móviles.

## Introduction

Globally, female sex workers (FSWs) are disproportionately affected by HIV and sexually transmitted infections, STI [1–6]. HIV prevalence among FSW is twelve times higher than the general population and, in some countries of Sub-Saharan Africa (e.g., Botswana, Kenya), as high as 37% [1, 7–9]. Universal HIV testing is an essential first step in the HIV treatment cascade [10–14]. World Health Organization (WHO) guidelines recommend that populations disproportionally affected by HIV, such as men who have sex with men (MSM) and FSW, get tested every 3–6 months [15, 16]. Many populations (e.g., FSW, MSM, migrants) in diverse settings face barriers to HIV testing due to stigma, criminalization, and limited access to health services [1, 14, 17–20]. However, less is known about the possible compounding effects of migration and mobility on HIV testing access [21, 22].

The Mexico–Guatemala border is at a key geographic position as a gateway for migration into Mexico and up to the United States [23–25]. Additionally, everyday commuters, seasonal agricultural workers, and truck drivers converge in this region [26–28]. In border cities, such as Tecun Uman, Guatemala, the mobile population is three times the resident population [28]. Given the anonymity that the influx of diverse mobile populations offers, the Mexico–Guatemala border is a also a major destination for FSWs [21, 28]. A qualitative study conducted with migrant FSWs in this region found that drivers of internal and international migration, such as violence in the community of origin from gangs, gender-based violence and the desire for improved economic opportunities, also push some women to engage in the sex work industry in this region [29].

HIV prevalence among sex workers is estimated at 4.5% in Guatemala and the prevalence of HIV, syphilis, chlamydia, and gonorrhea has been measured at 1.1%, 9%, 14%, and 12%, respectively, on the Mexico side of the border [21, 30]. Few studies have explored the association between migration and mobility and HIV risk among FSW in this region. An epidemiologic survey conducted in Guatemala found that in border cities, such as Tecun Uman and Malacatan, almost 40% of the surveyed FSWs reported engaging in sex work in at least two different cities in the past year [21].

A study of 2466 FSW in five Central American countries found that approximately 26% of participants were foreign-born, with 59% of the foreign born located in Guatemala [31]. Another study conducted in the Soconusco, a region close to the Mexican side of the border, found that this setting attracts Central American women to enter the sex trade industry, and there were high levels of STIs (i.e., a cumulative prevalence of treatable gonorrhea, chlamydia, and syphilis of 27.4%) [32]. Finally, a literature review on mobility and HIV in Central America and Mexico found that overall mobility is associated with increased HIV risk behaviors but may also be associated with preventive behaviors (e.g., increased condom use) [33]. Migration and mobility often intersect with other vulnerabilities, such as substance use, social exclusion and stigma, and limited access to prevention services, that may increase the odds of HIV transmission [33–35].

In both Mexico and Guatemala, sex work is quasi-regulated and tolerated in some zones. Public health practices surrounding sex work require that those who work in certain environments (e.g., formal sex work settings) undergo regular HIV/STI testing (i.e., every 3 months) at local clinics to maintain a health card (i.e., sex work registration) [36, 37]. In Guatemala, health permits are provided free-of-charge through community health clinics [36]. In Mexico, registration involves out-of-pocket fees and in Tapachula, Mexico, it additionally requires transportation to a clinic located in the tolerance zone, an isolated area within Tapachula (Las Huacas) [34].

Local police officers and immigration authorities along with health authorities frequently enforce public health regulations surrounding sex work (e.g., verify updated health cards) and often participate in raids on formal sex work settings [38]. Such practices give police and immigration authorities broad leverage to extort and unlawfully detain sex workers under the pretense of not maintaining their permits [38–40]. This mixing of public health promotion and involvement of legal authorities may have unintended consequences such as engendering a preference to work in less visible settings (e.g., street, hotels), workplace instability, and subsequently, not maintaining a health card or avoiding other interactions with HIV prevention services or local organizations (e.g., HIV prevention activities) regardless of the type of sex work setting [34, 38, 41–44]. Similar trends have been found in other settings such as the United States–Mexico border where characteristics such as visibility of venues and the likelihood of enforcement may explain FSW registration and condom accessibility [45].

In contrast to the aforementioned interactions, a quantitative study conducted in this border region found that migrant substance using FSW reported higher odds of consistent condom use with clients and having a health card in comparison to non-migrant FSW [35]. These findings are supported by a qualitative study conducted in the same setting which found that, regardless of migration status. Some of the FSW choose to have a health card in order to know their HIV status and to make sure they are healthy [38]. Other migrant FSW prefer to see private doctors or delay uptake of sexual and reproductive health services (SRH) until their next trip to their community of origin [34].

Overall, there is limited data on the accessibility of HIV testing and SRH services among FSW in this setting, and data on access to prevention services differ [21, 22]. A 2013 study conducted in six border regions including the Mexico–Guatemala border found that 61% of the 301 FSW reported receiving HIV testing and 57%, access to SRH services in the past 5 years [22]. An epidemiological survey conducted in Guatemala also in 2013 found that, in the border cities of Tecun Uman and Malacatan, 80% of the FSW reported receiving an HIV test in the past year [21]. It is important to highlight that access to health care services overall is limited in this setting. In the border state of Chiapas, one of the less developed states in Mexico, around 20% of the population does not have any access to health care services [46]. This is explained by the informality of the economy as well as other structural barriers, such as geographical distance to clinics and lack of personal identification documents [47]. Some studies have suggested that overall access to health care services may be especially challenging for migrants due to migration status and discrimination [48].

Given the migration context of the Mexico–Guatemala border and the nuances found in previous research conducted at this border, the aim of this paper is to determine if a specific form of migration and mobility (i.e., traveling to another country to engage in sex work) was associated with HIV testing in the past year among FSW in four communities along the Mexico–Guatemala border. Based on the literature and previous findings, we hypothesized that FSW who travel to another country to engage in sex work would have higher odds of reporting an HIV test in the past year compared to those who did not travel to engage in sex work [34, 49–51].

## Methods

### Study Setting and Procedures

From 2012 to 2015, we recruited FSWs (N = 266) as part of a cross-sectional study (*Cruzando Fronteras*) of substance use and HIV risk among key populations. HIV prevalence in this sample (3.4%) was described previously [35].

Using a combination of modified time-location sampling of sex work venues (e.g., bars, street) and peer referral, participants were recruited in the border cities of Ciudad Hidalgo and Tapachula in Mexico and Tecún Umán and Quetzaltenango in Guatemala. Eligibility criteria for the study included: (a) biologically female, (b) reported exchanging sex for money, drugs or goods in the last month, (c) illicit substance use (beyond marijuana) in the past 2 months, (d) 18 years old or older, (e) Spanish speaker, (f) willing and able to provide informed consent, and (g) willing to undergo on-site HIV testing. For the present study, we excluded 11 participants who did not report if they had ever received an HIV test or the date of their last test; thus, we analyzed the data of 255 participants. Upon written consent, trained interviewers administered face-to-face questionnaires to obtain information on sociodemographics, migration and mobility experiences, work environment factors, and substance use. Interviews were conducted in private rooms and lasted approximately 50–80 min. Participants were compensated $10 USD worth of in-kind goods for completing the interview and testing, and $5 USD for returning to receive their HIV test results. If positive, field staff accompanied participants to the local HIV/STI treatment institutions [i.e., Center for the Prevention and Treatment of HIV and Sexually Transmitted Infections (CAPASITS) in Mexico and the Health Ministry in Guatemala].

This project was approved by the involved institutions’ Human Research Protections Programs in Mexico, Guatemala and the U.S.

### Measures

#### Dependent Variable

Participants were asked if they had ever been tested for HIV before—not including testing associated with blood donation—and the date of their last HIV test. The variable HIV testing in the past year was built by calculating the months that had passed between the last HIV test and interview date. Participants were categorized as not having recent HIV testing if they had never been tested or had not been tested within the past year.

#### Exposure

*Travel to engage in sex work in another country* we created a dichotomous variable for whether or not participants reported engaging in sex work in another country besides where they were interviewed in the past year (yes/no). This illustrates mobility among FSW.

### Covariates

#### Sociodemographics

Included age, years in sex work, education, civil status, religious affiliation (including Catholic or Protestant/Evangelical Christian), and whether or not they have children. Income was dichotomized as earning more or less than 125 USD dollars per week (based on the 75th percentile).

#### Interaction with Local Health Services, Health Card

Participants were asked if they had an updated/current health card (yes/no) [38].

#### Type of Venue

Formal venues encompassed working in more ‘visible’ places, such as bars and nightclubs. In formal venues, sex workers need to have a health card, are subject to inspections by authorities, and usually have a manager or an owner in charge. Informal venues include working on the street or in hotels. Informal venues are clandestine and sometimes far from the tolerance zones, and women find their own clients and the place where they exchange sex. Some participants worked in more than one type of venue [29]. We also created a dichotomous variable for currently having to pay a pimp, manager, or bar owner as part of their earnings (yes/no). We did not ask participants about the nature of these third-party interactions (i.e., a range of experiences from supportive and protective to coercive and exploitative) [54]. We created a variable that captured participants who reported having more than 20 different clients in the past 30 days (based on the 75th percentile).

#### Substance Use

The Alcohol Use Disorders Identification Test Consumption (AUDIT-C) was used to assess problem drinking [55]. Additionally, categorical variables were created to reflect hazardous drinking for women: (a) drinking more than 4 days per week, and (b) drinking 4 or more drinks on a regular day. Participants were also asked if they had ever used drugs and how often during the last 6 months they used a certain drug. For analytical purposes, hard drug use comprised the use of cocaine, crack, crystal methamphetamine, or heroin by any mode of administration [56, 57]. Marijuana, inhalants, amphetamines, and tranquilizers were excluded from this definition.

### Data Analysis

**Fig. 1:**
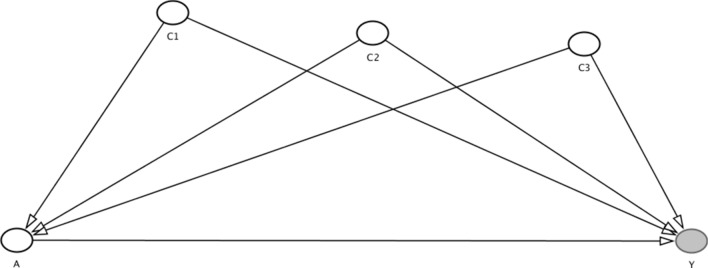
Directed acyclic graph (DAG) of traveling to another country to engage in sex work and HIV testing in the past year [53]

Our analysis was guided by the literature on FSW and the contrasting aforementioned patterns found in this setting. We used a directed acyclic graph (DAG) to illustrate the pathway that we believe is demonstrative of the association between the main predictor “traveling to another country to engage in sex work” (i.e., mobility in the context of sex work) and the outcome “HIV testing in the past year”. DAGs are non-parametric causal diagrams used to support causal analysis [52]. We also included the confounders that, based on the literature and our experience in this area, should be addressed in this analysis. In Fig. 1, A is our exposure (i.e., traveling to another country to engage in sex work) and Y is the outcome (i.e., HIV testing in the past year). Confounder C1 is sex work context characteristics (i.e., giving a percentage of earnings to the owner), confounder C2 is interaction with organizations that provide HIV prevention services, confounder C3 is interacting with local health services (i.e., health card).

Descriptive statistics were calculated to provide an overview of participants’ demographics: Pearson Chi-square or Fisher’s exact test for discrete variables and Wilcoxon rank sum for non-parametric continuous variables. Univariate logistic regressions were performed to characterize factors associated with recent HIV testing. Based on our DAG, we included confounders that impact both our main exposure (i.e., traveling to another country to engage in sex work) and the outcome (i.e., HIV testing in the past year) [52]. For the multivariate logistic regression, we included not only variables that were significant at p ≤ 0.05 but also those key in our DAG (e.g., sex work characteristics) [52, 58]. The paths illustrated in the DAG were tested to ensure the integrity of the model. All regression models are presented with crude and adjusted odds ratios and 95% confidence intervals, with any p ≤ 0.05 considered significant. Analyses were conducted with SPSS Statistics 21 Software (IBM, 2012).

## Results

Descriptive characteristics by mobility experience are summarized in Table 1. The median age of the sample was 27 years old [interquartile range (IQR) 24–37), the median years in sex work was 4 (IQR 2–11), and most of the sample (80%) was originally from Central America. Only 41% of the participants (n = 105) reported getting an HIV test in the past year.

**Table 1:** Descriptive characteristics of substance using female sex workers at the Mexico–Guatemala border by mobility experience (N = 255)

Characteristics	Travel to engage in sex work in another country (n = 53)	No travel to engage in sex work in another country (n = 202)	Total (n = 255)
	n (%)	n (%)	n (%)
HIV testing in the past year	35 (66)	70 (35)	105 (41)
Sociodemographics			
Age (median, IQR^a^)	29 (24–33)	26 (21–35)	27 (22–34)
Years in sex work (median, IQR^a^)	7 (3–13)	4 (1–9)	4 (2–11)
At least 1 year in sex work (median, IQR^a^)	49 (93)	175 (87)	224 (88)
Income (< 125 USD, weekly)	37 (70)	145 (72)	182 (71)
Marital status			
Not married	41 (77)	155 (77)	196 (77)
Has children	52 (98)	163 (81)	215 (84)
Of above, kids who are < 18 years old	49 (94)	153 (94)	202 (94)
Religion			
Maintains a religious affiliation	34 (64)	134 (66)	1168 (66)
Education			
Elementary school or less	39 (74)	106 (53)	145 (57)
Sex work characteristics			
Type of venue (n = 251)			
Formal venues^b^	19 (36)	61 (30)	80 (31)
Informal venues^c^	42 (79)	162 (80)	204 (80)
Gives a part of earnings to a bar owner or manager	21 (40)	52 (26)	73 (29)
Consistent condom use	25 (47)	84 (42)	109 (43)
Client volume (> 20, past month)	18 (34)	60 (30)	78 (31)
Interaction with organizations			
Participated in HIV/AIDS informational or educational activities, past year	23 (43)	38 (19)	61 (24)
Interaction with local health services			
Current health card	10 (36)	18 (9)	28 (11)
Substance use			
Drinks 4 or more days per week	14 (26)	61 (30)	75 (29)
Drinks more than 4 drinks on a regular day	43 (81)	172 (85)	215 (84)
Ever injection drug use (n = 9)	4 (7.5)	5 (2.5)	
Any hard substance use, past 6 months^d^	49 (93)	145 (72)	194 (76)
Country of origin			
Mexico	8 (15)	41 (20)	49 (19)
Guatemala	26 (49)	125 (62)	151 (59)
El Salvador	5 (9)	11 (5)	16 (6)
Nicaragua	0 (0)	10 (5)	10 (4)
Honduras	13 (25)	15 (7)	28 (11)
Dominican Republic	1 (2)	0 (0)	1 (0.5)

^a^Interquartile range

^b^Formal venues include reporting working in a bar, nightclub, *discoteque* or brothel. These venues usually require a health card

^c^Informal venues include reporting working n the street, *cantina*, *closed house* (clandestine space where women exchange sex with men), hotel, massage parlor, client’s car, private house, park or any other public space, place where they use or buy drugs

^d^Includes reporting the use of cocaine, crack, crystal meth, or heroin by any mode of administration

In the univariate analyses (Table 2), mobility was associated with increased HIV testing: 3.67 higher odds for those traveling to do sex work in another country and nearly twice the odds for recent as well as for frequent border crossers. Having a current health card (OR = 10.82, 95% CI = 3.63–32.26), working in formal venues (OR = 3.10, 95% CI = 1.79–5.37), and participating in HIV informational or educational activities (OR = 7.01, 95% CI = 3.63–13.6) were significantly associated with HIV testing in the past year. Giving a part of earnings to a bar owner or manager was associated with a nearly fivefold increase in the odds of HIV testing in the past year. High client volume (OR = 3.36, 95% CI = 1.94–5.86) was significantly associated with higher odds of HIV testing in the past year (Table 2). Finally, self-identifying as primarily being a housewife with occasional clients, working in informal venues, and earning less than 125 USD per week were all associated with lower odds of getting an HIV test in the past year.

**Table 2:** Univariate association with HIV testing in the past year among substance using female sex workers at the Mexico–Guatemala border (N = 255)

Characteristics	HIV testing in the past year (n = 105)	No HIV testing in the past year (n = 150)	OR (95% CI)	p value
	n (%)	n (%)		
Mobility				
Short-term travel to engage in sex work in another country, past year	35 (33)	18 (12)	3.67 (1.93–6.94)	** < 0.001**
Sex work characteristics				
Type of venue (n = 251)^a^				
Formal venues^b^	48 (47)	32 (22)	3.10 (1.79–5.37)	** < 0.001**
Informal venues^c^	76 (74)	128 (87)	0.45 (0.24–0.84)	** < 0.05**
Gives a part of earnings to a bar owner or manager	49 (47)	24 (16)	4.59 (2.57–8.21)	** < 0.001**
Consistent condom use	62 (59)	47 (31)	3.16 (1.87–5.31)	** < 0.001**
Client volume (> 20, past month)	48 (46)	30 (20)	3.36 (1.94–5.86)	** < 0.001**
Interaction with organizations				
Participated in HIV/AIDS informational or educational activities, past year	46 (45)	15 (10)	7.01 (3.63–13.6)	** < 0.001**
Interaction with local health services				
Current health card	24 (23)	4 (3)	10.82 (3.63–32.26)	** < 0.001**
Individual level				
At least 1 year in sex work	94 (90)	130 (87)	0.49 (0.60–2.87)	0.493
Self-identified as a street worker	26 (25)	34 (23)	1.12 (0.63–2.02)	0.698
Self-identified as a bar sex worker	37 (35)	21 (14)	3.34 (1.82–6.16)	** < 0.001**
Self-identified as a housewife with occasional clients	15 (14)	51 (34)	0.32 (0.17–0.62)	** < 0.001**
Self-identified as a *fichera*^d^	17 (16)	8 (5)	3.43 (1.42–8.35)	** < 0.001**
Substance use				
Drinks 4 or more days per week	35 (33)	40 (27)	1.38 (0.79–2.37)	0.251
Drinks more than 4 drinks on a regular day	93 (89)	122 (81)	1.78 (0.86–3.68)	0.121
Ever injection drug use (n = 9)	6 (6)	3 (2)	2.97 (0.73–12.15)	0.167
Any hard substance use, past 6 months^e^	88 (84)	106 (71)	2.15 (1.15–4.02)	** < 0.05**

^a^Some participants reported working in more than one place as their main workplace and some of them reported a formal and an informal venue as their main workplace

^b^Formal venues include reporting working in a bar, nightclub, *discoteque* or brothel. These venues usually require a health card

^c^Informal venues include reporting working in the street, *cantina*, *closed house* (clandestine space where women exchange sex with men), hotel, massage parlor, client’s car, private house, park or any other public space, place where they use or buy drugs

^d^*Ficheras* are women who usually drink alcoholic or non-alcoholic beverages with clients. For each drink paid by a male client they receive a token (fichas in Spanish) and at the end of the day the bar owners give cash in exchange for the tokens obtained.

^e^Includes reporting the use of cocaine, crack, crystal meth, or heroin by any mode of administration. Boldface indicates characteristics that were statistically significantly associated at p < 0.05 with HIV testing in the past year

### Multivariate Model

Based on the literature and our previous work in this setting, we created a DAG with relevant covariates that could be contributing to the variability of the pathway between the main exposure (i.e., mobility) and the outcome HIV testing in the past year [52]. Given the DAG (Fig. 1), we included in a multivariate logistic regression model the main exposure (i.e., traveling to engage in sex work) and relevant confounders that included sex work characteristics (i.e., give a part of earnings to a bar owner or manager), interaction with organizations (i.e., participation in HIV prevention activities), interaction with local health services (i.e., current health card) and working at least 1 year in the sex industry (Table 3). We found that those who travel to engage in sex work in another country had significantly higher odds of HIV testing in the past year considering the aforementioned confounders (AOR = 2.34, 95% CI = 1.13–4.85).

**Table 3:** Multivariate logistic regression of mobility and HIV testing in the past year among substance using female sex workers at the Mexico–Guatemala border (N = 255)

Characteristics	AOR (95% CI)	p value
Mobility		
Travel to engage in sex work in another country, past year	2.34 (1.13–4.85)	< 0.05
Cofounders		
Sex work characteristics		
Give a part of earnings to a bar owner or manager	2.55 (1.31–4.97)	** < 0.001**
Interaction with organizations		
Participation in HIV/AIDS informational or educational activities, past year	4.90 (2.39–10.04)	** < 0.001**
Interaction with local health services		
Current health card	7.24 (2.25–23.25)	** < 0.001**

## Discussion

HIV testing in the past year occurred among 41% of this population of FSWs. This is especially low considering that WHO recommends sex workers access voluntary HIV testing every 3–6 months [16]. We found that FSWs who traveled to another country for sex work had higher odds of being tested for HIV in the past year compared to those who did not travel to another country to do sex work. Prior work in this region has found that international migrants reported higher levels of consistent condom use and health card ownership, entailing high levels of HIV/STI testing, compared to non-international migrant sex workers [59]. These findings suggest that perhaps mobile women among this sample are being better reached by public health services than women who do not travel.

A previous qualitative study conducted among sex workers at the Mexico–Guatemala border found that international migrants engaged in circular migration (i.e., to their home community or country) to access certain SRH services that were not accessible in the interview site [34]. This may be happening among the participants who reported traveling to another country to engage in sex work.

Another factor to take into consideration is that we found that high client volume (more than 20 different clients, past month) was associated with HIV testing in the past year in the univariate analysis. Some studies have found that risk perception may play an important role in health seeking behaviors [51, 60]. It is possible that exposure to risky sexual behaviors, such as having many different clients, may motivate women to get tested. Furthermore, high client volume, as well as giving a part of earnings to a bar manager or owner, may be correlated with the type of sex work venue or its characteristics. A previous analysis from this study found that substance using international FSW women had higher odds of reporting high client volume but also had more consistent condom use [35].

It is important to consider the characteristics of the participants who did not receive an HIV test in the past year. In the univariate analysis, participants who reported work in informal venues had lower odds of receiving an HIV test (Table 2). Studies conducted in border settings have found that informal and unstable workplaces are associated with reduced access to health services and less interaction with local organizations [61, 62]. An interesting variable to consider are women who self-identify as a housewife, but occasionally engage in sex work: 35% of the women who did not receive an HIV test in the past year fell into this category. In the crude analysis, this was significantly associated with lower odds of getting an HIV test. As mentioned previously, not identifying as a sex worker (but just occasionally doing sex work on the side) and broader structural conditions (e.g., sex work venue, stigma) may result in inadequate access to prevention services and interaction with local organizations [63, 64].

This study has several limitations. The data used for this analysis are cross-sectional; thus, causality may not be inferred. Data were self-reported; therefore, social desirability or recall bias may have influenced some results. In order to address this potential bias, trained local staff conducted all the interviews in safe, private spaces (e.g., study office) after conducting extensive outreach to establish trust and explaining in detail what we meant by “ever HIV testing.” Participants also were assured that their individual responses would be kept confidential and would not affect their current access to care or services. As the data were collected via modified time-location sampling and peer-referral, we are not able to generalize to all sex workers in this or other settings. Nevertheless, this analysis contributes to understanding the limitations of HIV testing among populations disproportionally affected by HIV.

## Conclusions

This study provides evidence that overall access to HIV testing among FSWs in border settings needs to increase. Efforts especially need to consider voluntary and non-stigmatizing prevention HIV services and focus on reaching out to less mobile women. FSWs who reported traveling to engage in sex work had higher odds of receiving an HIV test. This might warrant further research to understand the structural conditions for why mobile FSWs have increased access to HIV testing or if risk perception is playing an important role in such an association. As part of broader multi-prolonged HIV prevention interventions, sex worker communities and local organizations should be consulted when designing HIV/STI prevention campaigns and programs [40, 65].

## Data Availability

Not applicable. Not applicable.
